# Microevolutionary, macroevolutionary, ecological and taxonomical implications of punctuational theories of adaptive evolution

**DOI:** 10.1186/1745-6150-8-1

**Published:** 2013-01-16

**Authors:** Jaroslav Flegr

**Affiliations:** 1 Jaroslav Flegr, Faculty of Science, Charles University in Prague, Viničná 7, CZ-12844, Praha 2, Czech Republic

**Keywords:** Speciation, Frozen plasticity, Frozen evolution, Peripatric speciation, Invasive species, Domestication, Asexual species, Genetic draft, Genetic hitchhiking, Advantage of sex, Evolutionary trends, Dead clade walking, Cambrian explosion, Origin of genera, Taxonomy

## Abstract

**Abstract:**

Punctuational theories of evolution suggest that adaptive evolution proceeds mostly, or even entirely, in the distinct periods of existence of a particular species. The mechanisms of this punctuated nature of evolution suggested by the various theories differ. Therefore the predictions of particular theories concerning various evolutionary phenomena also differ.

Punctuational theories can be subdivided into five classes, which differ in their mechanism and their evolutionary and ecological implications. For example, the transilience model of Templeton (class III), genetic revolution model of Mayr (class IV) or the frozen plasticity theory of Flegr (class V), suggests that adaptive evolution in sexual species is operative shortly after the emergence of a species by peripatric speciation – while it is evolutionary plastic. To a major degree, i.e. throughout 98-99% of their existence, sexual species are evolutionarily frozen (class III) or elastic (class IV and V) on a microevolutionary time scale and evolutionarily frozen on a macroevolutionary time scale and can only wait for extinction, or the highly improbable return of a population segment to the plastic state due to peripatric speciation.

The punctuational theories have many evolutionary and ecological implications. Most of these predictions could be tested empirically, and should be analyzed in greater depth theoretically. The punctuational theories offer many new predictions that need to be tested, but also provide explanations for a much broader spectrum of known biological phenomena than classical gradualistic evolutionary theories.

**Reviewers:**

This article was reviewed by Claus Wilke, Pierre Pantarotti and David Penny (nominated by Anthony Poole).

## Open peer review

Reviewed by Claus Wilke, Pierre Pantarotti and David Penny (nominated by Anthony Poole). For the full reviews, please go to the Reviewers' comments section.

## Background

**Figure 1:**
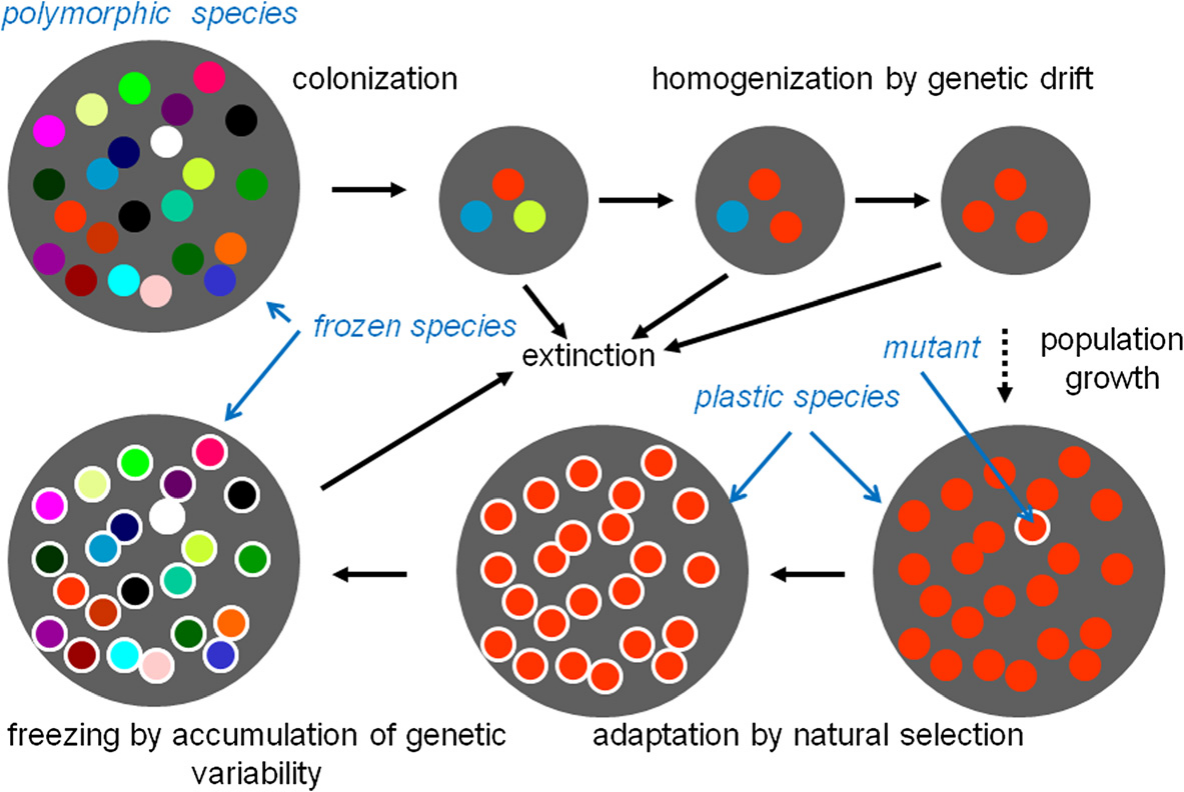
**Adaptive evolution in sexual species according to frozen plasticity theory.** It must be emphasized that extinction is a far more probable fate for a small population than expansion. However, unsuccessful speciation events are not interesting from the perspective of evolution.

Most punctuational theories of evolution, including the evolutionary conceptions of Wright, Mayr, Carson, Templeton and Flegr (for comparison see Table 1), suggest that sexually reproducing species respond evolutionarily to selection (are evolutionarily plastic) only during speciation. The mechanisms of this type of evolutionary behavior of sexual species suggested by the various theories differ, for a review see [1]. For example, the genetic revolution model [2] implicitly and the frozen plasticity theory explicitly [3] suggest that a species is evolutionary plastic when its members are genetically uniform, i.e. only after a portion of the original species has split off, skirted extinction for several generations, and then undergone rapid multiplication (Figure 1).

**Table 1:** Differences between various punctuational theories and models

**Theory and its author**	**The aim**	**Suggested mechanism**
Shifting balance theory	to explain the ability of species with large subdivided populations cross valleys in adaptive landscape	1. fragmentation of population to small subpopulations where an efficiency of selection is low 2. spreading and fixation of a new allele (that is detrimental when rare) in a subpopulation by drift 3. “Infection” of other subpopulations with individuals with new genotype originated from a successful population and the origination of new populations by these individuals
Wright S. 1932 ^1^		
Genetic revolution	to explain the role of founder events in speciation	1. change of balanced frequency of alleles in a split-off subpopulation due to sampling effect 2. selection for alleles with best effect on fitness instead of best-cooperator alleles
Mayr E. 1954 ^2^		
Founder-flush model	to explain the role of founder events in speciation	1. sampling effect due to rapid one-step reduction of a population size, 2. expansion of the population in an open uninhibited ecological niche, which relaxes all forms of selection allowing for surviving recombinants and mutants with suboptimal phenotypes (crossing valleys in the adaptive landscape) 3. reaching (or overshooting) the carrying capacity of a locality and the restoration of selection
Carson H.L. 1968 ^3^		
Genetic transilience model	to explain the role of founder events in speciation	1. sampling effect due to rapid one-step reduction of a population or to hybridization, 2. an increase of the amount of selectable genetic variability due to transformation of nonadditive (and therefore nonselectable) genetic variability to additive genetic variability and by higher survival probability for carriers of new mutations in the expanding population, which increases responsiveness of the population to selection 3. restoration of the population size limitation and selection
Templeton A.R. 1980 ^4^		
Punctuated equilibrium	to explain the discontinuous nature of evolution and coincidence of anagenetic and cladogenetic events	various mechanisms suggested by Eldredge and Gold, including peripatric speciation and strong selection in unusual conditions on the periphery of the species’ range, peripatric speciation accompanied by genetic revolution, sorting (according to Futuyma^7^, without speciation, any evolutionary novelty is reversible due to gene flow), etc.
Eldredge N. 1971 ^5^		
Frozen plasticity theory	to explain why old species are microevolutionarily elastic and macroevolutionarily frozen, how frozen species can turn plastic, and the continuously decreasing rate of macroevolution	1. most polymorphism existing in an old species is sustained in it’s gene pool by frequency dependent selection creating interconnected network resistant to changes of allele frequencies, 2. most new (potentially useful) alleles are captured in this elastic network of alleles due to pleiotropy and its effect on (stabilized) frequencies of old alleles, 3. in small split-off populations balancing on the edge of extinction for several generations, a decrease in strength of selection, including frequency dependent selection, will occur, and most genetic polymorphism will disappear due to drift 4. after expansion of population size, now large genetically uniform population turns evolutionary plastic – new advantageous mutations can spread in the network-free population by selection 5. traits resistant to thawing accumulate in the gene pool by sorting on the basis of stability 6. accumulation of permanently frozen traits by the mechanism of sorting on the basis of stability in particular clades during macroevolution
Flegr J. 1998 ^6^		

The Genetic Revolution model implicitly and the Frozen Plasticity model explicitly suggest that frequency-dependent selection plays an important role in stabilization of the gene pool of a species. Therefore, according to these two theories, macroevolutionary frozen species are microevolutionarily elastic. According to the Shifting Balance theory, the Founder Flush and the Genetic Transilience models, they are microevolutionary frozen, i.e. they have significantly reduced plasticity in comparison with their plastic state. According to Futuyma’s sorting model, macroevolutionarily frozen species can be microevolutionarily plastic. ^1^ (Wright 1932), ^2^ (Mayr 1954), ^3^ (Carson 1968), ^4^ (Templeton 1980), ^5^ (Eldredge 1971), ^6^ (Flegr 1998), ^7^ in (Gould 2002), p. 77. In fact, the Punctuated equilibrium theory in its current form was published in 1972 by Eldredge and Gould (Eldredge and Gould 1972) and the Frozen plasticity theory in 2008 (Flegr 2008) and 2010 (Flegr 2010) by Flegr.

Following a short period of time, estimated by paleontological data to correspond to 1-2% of the existence of the species, polymorphism accumulates in the gene pool; and thus, in each generation, new mutations occur in the presence of different alleles. The theory of evolutionarily stable strategies indicates that, under these conditions, selection cannot lead to long-term changes in the phenotypes of organisms (an analogy of the fixation of the “best” strategy in a population), but only to a deflection of the frequency of the individual alleles (strategies) from evolutionarily stable equilibrium. The greater this deflection, the more the gene pool resists the selection; after it ceases, the allele frequencies spontaneously return to their original values. Therefore, the species ceases to be evolutionarily plastic and becomes elastic on a microevolutionary time scale and frozen on a macroevolutionary time scale. It then exists in this state until (unfavorable) environmental changes accumulate so that the macroevolutionarily frozen species becomes extinct.

Here I show that punctuational models of evolution have considerable evolutionary and ecological implications (see Table 2) that could be tested empirically, and should be analyzed theoretically in greater depth.

**Table 2:** Differences between predictions of the gradualistic and punctuational theories of evolution

	**Gradualistic theories**	**Group I punctuational theories**	**Group II punctuational theories**	**Group III punctuational theories**	**Group IV punctuational theories**	**Group V punctuational theories**
typical representative	Fisher’s model	Futuyma’s model	Wright’s model	Templeton’s model	Mayr’s model	Flegr’s model
anagenesis and cladogenesis are coupled **^1, 2^	no	yes	yes	yes	yes	yes
divergence of species correlates with taxon richness ^1^	no	yes	yes	yes	yes	yes
genetic polymorphism decelerates evolution **^3^	no	no	no	no	yes	yes
most species under usual conditions respond to selection *^4^	as plasiticine	as plasiticine	rather as lead than plasticine	rather as lead than plasticine	as rubber	as ruber
two species in the same niche frequently can easily coexist *	no	no	yes	yes	yes	yes
species are adapted to original environment *^5^	no	no	yes	yes	yes	yes
local and global abundance do not correlate for old species **^6^	no	no	yes	yes	yes	yes
abundance of species decreases with species age	no	no	yes	yes	yes	yes
ability of species to respond to environmental changes decreases with species age **^7^	no	no	no	no	yes	yes
ability of species to change taxon-characteristic traits decreases with clade age *	no	no	no	no	no	yes
species on islands are derived more than those on continents *^1^	no	yes	yes	yes	yes	yes
asexual species are more adapted to their environment *^8^	no	no	no	yes	yes	yes
cross-pollinating species more stable than self-pollinating species *^9^	no	yes	no	yes	yes	yes
invasive species express higher capacity to respond selection **^10^	no	no	no	yes	yes	yes
domesticated species express higher capacity to respond selection	no	no	no	yes	yes	yes
domesticated species are evolutionarily younger	no	no	no	no	yes	yes
successful selection decreases fitness *^11^	no	no	no	no	yes	yes
evolution of altruistic behavior by group selection is easy *^12^	no	no	no	yes	yes	yes
phylogenetic trees usually resemble *^13^	tree	shrub	shrub	shrub	shrub	shrub
intraspecies variability in a clade usually decreases in time*	no	no	no	no	no	yes
interspecies variability (disparity) in a clade usually decreases *	no	no	no	no	no	yes
dead clade walking should frequently occur *	no	no	no	no	no	yes
slow long-term trends are quite possible *	no	yes	yes	yes	yes	yes
genera and higher taxa are objective existing entities *	no	yes	yes	yes	yes	yes

Gradualistic theories include not only classical neodarwinistic (Fisherian) models but also selfish gene model of Dawkins (Dawkins 1976). The Futuyma’s model (stabilization of gradualistically developed traits by a speciation) was described in (Gould 2002), p. 77, other models are described in Tab. 1. The group II encloses the Wright’s Shifting balance model (Wright 1932) and the group III encloses the models of Carson and Templeton (Carson 1968; Templeton 1980) as the elasticity of species or the frequency dependent selection is probably not explicitly mentioned in these models. The Flegr’s frozen plasticity model (class V) differs from the Mayer’s Genetic revolution-based model (class IV) by including theory of evolutionary stable strategies for description of behavior of alleles in genetically polymorphic population and by including the conception of accumulation of permanently frozen traits by sorting for stability. Two asterisks denote the predictions that have already been tested with positive result. One asterisk denotes the predictions that have not been intentionally tested but are supported by published data. ^1^ (Ricklefs 2004), ^2^(Pagel *et al.* 2006), ^3^(Bryant *et al.* 1986; Mezhzherin 1997), ^4^ (Dobzhansky and Spassky 1969), ^5^(Costas *et al.* 1996), ^6^(Prinzing *et al.* 2004), ^7^(Mikulas 2008), ^8^(Haag and Ebert 2004; Peck *et al.* 1998), ^9^(Flegr 2002), ^10^ (Novak 2007; Prentis *et al.* 2008; Yonekura *et al.* 2007), ^11^(Bradshaw and Holzapfel 2006; Nussey *et al.* 2005), ^12^ (Kulich and Flegr 2010), ^13^ (Gould 2002; Heard 1992).

## Results and discussion

### Microevolutionary elasticity and adaptation to past condition

According to the classical gradualistic theories, all species respond to selection as if they were plasticine while, according to punctuational theories, most species are resistant to selection as if they were lead (class II and class III theories) or respond to selection as though they were rubber – at first, they respond readily to selection pressure; however, as the average phenotype of the organism deviates from its original state, selection is less and less effective and, at a certain point, the response ceases (class IV or class V theories) (Table 2). According to class IV and class V punctuational theories, the average phenotype returns to the original state when the selection stops [3].

There are several critical implications: in the world of species that do not respond to selection, organisms are not optimally adapted to the conditions of their current environment but to those present during the evolutionary plasticity of the particular species. This should be true especially for evolutionarily old species, as their environmental conditions probably differ most from those existing during their origination. For example, algae (the typical representatives of ultrabradytelic species), which originated in the Paleozoic when days lasted about 21 hours, are known to better synchronize their circadian rhythms with shorter light–dark cycles than the current 24-hour cycle [4].

### Lower Viability and Fertility of Selected Organisms

Representatives of old, either microevolutionary frozen and therefore “obsolete” species (class II and III theories) or elastic species, kept out of their original state by selection (class IV and V theories), have lowered viability or fertility in comparison with representatives of young species living under conditions similar to those existing at the time of their origination [3]. Therefore, the population density is probably negatively correlated with species age; a study of the correlation of the molecular age of a species with its average abundance could easily test this prediction.

The correlation could also explain the existence of the most universal ecological law – that every community shows a hollow curve on a histogram with many rare, and only a few common species [5]. This is a quite stable situation; species retain their basic status as common or rare for millions of years [6]. Class II-V punctuational theories of evolution predict that common species are young species, still evolutionarily plastic or having recently lost their plasticity, that still live under conditions similar to those existing at the time of their origination. This agrees with the observed correlation between global and local abundance in young species, but not in old. Old species are probably less competitive in a similarly broad spectrum of biotopes as young species [7]. Class II-V punctuational theories of evolution also predict that the paleontological record will more often show a gradual change from common to rare species rather than the opposite change from rare to common species.

### Limited Geographical Range of Species

On the basis of class IV and class V theories, it can also be expected that populations near the center of the species’ range express higher mean viability or fertility than those on its periphery, which have had to adapt to conditions different from those at the time of its origination. E.g. tits are able to adapt to a different climate, with its corresponding shift in peak abundance of caterpillars, by a shift of their own breeding season. However, the fertility of these adapted populations decreases in comparison with birds adapted to the original climate [8,9].

Negative correlation between deviation from the equilibrium frequency of alleles (and from the original phenotype of the species) and the fitness predicted by any class IV and class V punctuational theory of evolution could provide an alternative explanation for the existence of distinct geographic ranges of species. Elastic species can adapt to geographically changing conditions only to a certain degree. At some point, the decrease in fitness accompanying the departure of the phenotype from the original state is so great that it is incompatible with the long-term survival of the population.

### Lower Viability and Fertility of Decorative Breeds

The same negative correlation between departure of a phenotype from the original state and the mean fitness could explain the lower viability and fertility of most decorative breeds of practically any domesticated species. When the populations of pure-bred animals are left to their fate, members of the population return to the phenotype of their wild predecessors within a few generations. This phenomenon differs from the return of the phenotype to an original wild form in the case of crosses between two different races. In crosses, the almost immediate return to the original phenotype is caused by a breakdown of the unique combination of alleles (responsible for the appearance of the members of the individual races) as a consequence of recombination and segregation of alleles. In members of the same race, there is a gradual return to the wild phenotype as a consequence of the action of natural selection which, during a few subsequent generations, removes from the population the individuals with reduced viability and fertility, i.e. with the phenotype of the human-bred race.

### Coexistence of Species that Use the Same Resource

The absence of evolutionary plasticity predicted by class II, III, IV and V punctuational theories could also explain long-term coexistence of species that use the same resource. Theoretical analysis shows this coexistence is possible, but highly unstable in evolutionarily plastic species [10]. Sooner or later, one of them increases in exploitation intensity or efficiency, thereby causing the extinction of the competing species. The absence of evolutionary plasticity in sexual species could be an important positive factor in the conservation of global and local biodiversity.

### Efficiency of Group Selection in Non-plastic Species

The low and vanishing inheritance of phenotypic traits in polymorphic sexual species predicted by class III, IV and V punctuational theories could also explain the persistence of altruistic behavior and general efficiency of group selection. The most serious objection of evolutionary biologists against the role of group selection in evolutionary processes consists in the fact that a trait that provides an advantage to a group and simultaneously places the individual that is its carrier at a disadvantage has a low chance of spreading and enduring in nature. Groups in which the altruistic trait spreads would prosper better than groups in which this trait is lacking and the average fitness of the members of this group would be greater; however, selfish individuals who do not exhibit this trait and do not behave altruistically, but only enjoy the advantages provided by the presence of altruists, would have the greatest fitness within these groups. In sexual (elastic) species, any behavioral trait (for example, altruistic behavior) is usually determined by the greater number of genes and many of these genes have (due to epistasis) a context-dependent influence on the particular trait. Consequently the heritability of most traits is low. Under these conditions, altruists emerge from the population as if by chance in families that are completely unrelated and have different phenotypes, i.e. individuals with quite different behavior, with a probability that is determined only by the proportion of particular alleles in the entire population. Thus populations can compete for the greatest average fitness of their members; those that have the greatest proportion of the relevant alleles, resulting in the greatest number of altruists being formed (emerging by chance), will win in this competition. The models show that group and inter-species selection can occur in nature in favor of altruistic traits (because the percentage proportion of alleles in the population is inherited from one generation to the next) and its results cannot be cancelled out by individual selection because the trait itself, altruistic behavior, is not inherited [11].

### Existence and Success of Invasive Species

The existence of two species types, very common non-plastic (microevolutionary frozen, according to class II and III theories or elastic, according to class IV and V theories) and very rare plastic, offers a new explanation for the existence of invasive species. The transfer of a species to a new territory is a necessary, but not sufficient, condition for invasion. In the vast majority of cases, the species succumbs to competition with local species and dies out. Only a small fraction of introductions “succeed”. For example, red deer were introduced into New Zealand a total of 32 times and only the last attempt was successful; however, these deer now occupy the entire area of the southern island [12]. Similarly, the now excessively successful starling settled in America only after at least nine attempts [13]. Invasive success is usually preceded by a relatively long lag phase, in which the future invasive species peacefully coexists with native species in the limited area of their original introduction.

According to classical gradualistic evolutionary theories, native species, which are adapted to local conditions, should outcompete newly introduced species [14,15]. According to the discussed class II-V punctuational theories, the ecological success of some newcomers is not very surprising. During the introduction and lag phase, the genetic polymorphism of an introduced population decreases, which could result in the conversion of a non-plastic species to the plastic state [16]. Non-plastic species are best adapted to the conditions existing at the time of their origin (past conditions), while plastic species can adapt to current conditions. Moreover, plastic species can outcompete non-plastic species in the coevolutionary arms-race.

Data on the evolutionary plasticity (evolvability) of invasive species are rather scarce [17-19]; however, e.g., the invasive grass *Phalaris arundinacea* demonstrates greater heritability and higher evolutionary plasticity (greater response of the phenotype to the local conditions) in North America than in its original area in Europe [20]. In accordance with the predictions of punctuational theories, parthenogenetic species (which always have much greater heritability of fitness than sexual species) [21] and polyploid species (which have often slipped through a genetic bottleneck as species of peripatric origin) [19] are over-represented among invasive species.

### Low Efficiency of Domestication of Plant and Animal Species

The existence of only a low proportion of evolutionarily plastic species can also explain the fact that humans have succeeded in domesticating only a negligible number of plant and animal species [22]. Only plastic species can adapt to the drastically changed conditions of life in captivity without a substantial reduction in viability and fertility. Class III, IV and V punctuational theories of evolution explicitly or implicitly suggest that domestication should be successful mostly in young, unfrozen species. It is worth recalling that most selection experiments were performed either on domestic animals, probably with lower genetic variance from the very beginning, [22] or on small populations that had passed through a narrow bottleneck just before, or at the beginning of, the experiment. Therefore, the ability of a species to respond to selection is probably overestimated and the natural elasticity is underestimated by the results of these experiments or of long-term selection programs performed on domesticates [23].

Class III, IV and V punctuational theories predict that most varieties of domesticated plants would have been derived from species with a capacity for vegetative reproduction, e.g. by means of tubers, rhizomes or grafts, or from self-pollinating species [22]. The plasticity of asexual species is higher than that of sexual species, and that plasticity is greater in self-pollinating species than in cross-pollinating species [24]. Therefore, these species can be more readily changed by artificial selection. On the other hand, sexually reproducing and cross-pollinating varieties should be more stable and lose properties acquired by artificial selection more slowly. Due to natural selection, a plastic variety has a tendency to increase its fertility at the expense of properties useful for man. In contrast, a sexually reproducing (elastic) variety can only respond to selection to a certain degree, and therefore cannot lose its useful properties due to natural selection. It was reported in the older literature that the varieties of cross-pollinating rye usually remained in seed company catalogues much longer than did those of self-pollinating wheat [24].

### Success of Asexual Species in Habitats with Extreme Conditions

The plasticity of asexual species should be greater in habitats that are poor in resources or where survival is limited by unfavorable abiotic factors. Here, the main criterion of evolutionary success is how well (not how quickly) the species can change its phenotype in response to environmental requirements. It is noteworthy that asexual species or asexual lineages of otherwise sexual species are found primarily in habitats with extreme conditions – in habitats that are extremely dry, cold or poisonous. The proportion of asexual species increases, for example, with increasing altitude and latitude, or where the soil contains high concentrations of poisonous heavy metals [25,26]. On the other hand, elastic sexual species should be better off in an environment rich in resources and with many competing species where the rate of evolutionary responses in the coevolutionary arm-race plays the crucial role. The fact that they retain most of their genetic polymorphism enables them to rapidly respond to any selection pressures by shifting the frequencies of their alleles without needing to wait for rare advantageous mutations.

### Evolutionary passivity of elastic species and the advantage of sex

Elasticity of sexual species predicted by class IV and V theories or evolutionary passivity of sexual species predicted by class III theories could also be advantageous in a long-term perspective. Under the fluctuating conditions of a stochastic environment, plastic asexual species could adapt to transient environmental change while non-plastic species resist such a change of their phenotypes. When the environmental conditions return to normal, a plastic species could fail to return to its optimal phenotype rapidly enough to avoid the risk of extinction, while the population of an elastic species (class IV and V theories) returns to its original phenotype within a few generations and a population of microevolutionary frozen species (class III theories) stays near the original optimum all the time. As suggested by G.C. Williams [27], the main advantage provided by sexual reproduction could consist in a substantial reduction in the evolutionary capability of sexual species. As a consequence of their elasticity and/or frozenness, sexual species are evolutionarily passive throughout much of their existence and cannot opportunistically respond to temporary short-term changes in the external conditions.

### Coincidence of Changes of the Phenotype of Organisms with Speciation

According to gradualistic models, there should be no correlation between cladogenesis and anagenesis (between speciation and changes in the phenotype of organisms) while punctuational models of any class assume that major irreversible phenotypic changes are always associated with speciation. The opposite does not hold, as most speciation events, such as vicariant allopatric speciation, parapatric speciation and many forms of sympatric speciation, are not coupled with a dramatic reduction in genetic polymorphism and return to plasticity. These forms of speciation could be responsible for the origin of most species, while new genera or higher taxa (i.e. monophyletic lineages with characteristic prominent evolutionary novelties) mostly result from peripatric speciation. Therefore, punctuational theories of evolution predict that the number of evolutionary changes in phenotype in a phylogenetic lineage reflects the number of speciations in this line rather than its age. A study of passerine birds has found the number of speciation within a phylogenetic line to have a very strong effect on the rate of anagenesis. The number of species alone explained 33.3% of the total variation in morphology [28]. Moreover, the reported rate of anagenesis on islands seems to be higher than on the mainland [28]. The higher frequency of peripatric speciation on islands can be a clue for explanation of the observed phenomenon.

Another corollary of the anagenesis-cladogenesis association predicted by the punctualistic models of evolution is that the extant representatives of ancient phylogenetic tree branches that have sustained a lower number of speciation events should bear more plesiomorphic characters than representatives of apical branches of the phylogenetic tree. According to classical gradualistic theories of evolution, no such correlation between species age and its antiquity should be expected.

### Correlation between the Rate of Molecular Evolution and the Speciation Rate

The correlation between the rates of anagenesis and speciation can be detected even on a molecular level. A molecular study [29] has shown that a relatively large part of the variability in the substitution rate can be explained by differences in the speciation rate between evolutionary lineages. Of course, a large part of the monitored nucleotide substitutions are neutral mutations known to be fixed by means of genetic drift and genetic draft and not by selection. Drift probably operates at the same rate in frozen, elastic and plastic species, however, the genetic draft operates more effectively during plastic phase of evolution when many neutral and nearly neutral mutations are being fixed with positive mutations by genetic hitchhiking. Approximately 35% of the substitutions (20-70%, depending on the studied taxon) was shown to occur in brief periods of speciation. It is worth mentioning that we are not aware of how many speciation events actually occur in the studied, seemingly unbranched lineages. Therefore, the published estimates of speciation-associated substitution rates represent only the lower margin of the real figures.

Molecular studies also confirm increased rates of evolution in island species. These species have not only a higher substitution rate but also a higher frequency of nonsynonymous substitution among the observed mutations, which suggests that positive selection rather than drift plays a more important role on islands (where a higher frequency of peripatric speciation is expected) [30]. Of course, another explanation for observed higher nonsynonymous substitution rate in island species, namely the higher probability of fixation of slightly negative mutations during peripatric speciation, also exists.

### Punctuational Evolution and the Origin of Evolutionary Trends

The class II-V punctuational models of evolution also offer a new explanation for the existence of evolutionary trends, the slow directional phenotypic changes in organisms of particular phylogenetic lineages that endure much longer than the individual species involved. The trends are too slow to be geared by selection – the change in the value of the trait per generation is so small that it is completely invisible for selection [31], p. 835. According to gradualistic evolutionary theories, the selection pressure has to be sufficiently strong to overcome genetic drift. However, this type of selection should result in far more rapid changes than those that emerge as trends in the paleontological record. Punctuational theories suggest a new solution to the paradox of very slow evolutionary trends. According to punctuational theories, the trend could, in fact, be a product of a relatively strong and long-term selective pressure to which species can respond, however, only in the brief and rare periods of their evolutionary plasticity.

### Shrub-Shaped rather than Tree-Shaped Phylogenetic Trees

Long-term, the number of species on Earth is relatively stable or even increases [32-35]. Thus, if some species become extinct without speciation, then other species must necessarily undergo speciation a great many times. It is therefore highly probable that a species in a transiently plastic state splits off not one but several different species. It has already been pointed out that the shape of phylogenetic trees differs significantly from that predicted by the neutral model of random speciation and extinction [31,36]. Phylogenetic trees are usually shrub-shaped rather than tree-shaped. Most disparate species originate simultaneously from a common ancestor as a result of adaptive radiation. Particular species that have originated in a common radiation event and from a single evolutionarily plastic ancestor coexist for a long time, without splitting off new species. Most branches end without producing a successor; however, some of them could split off a new plastic species that could undergo a new burst of radiation. Interestingly, such a tree is similar in shape to the figure drawn by Darwin [37] and unlike modern trees (which are usually automatically interpreted as phylogenetic trees but are in fact inspired by the shape of the cladogram, a graphic representation of the distribution of synapomorphies within a taxon).

### Higher Variability of Early-Branched Species and Decreasing Speciation Rate of Clades

The decreased variability of species with age of the phylogenetic line and the maximum biodiversity achieved early after the origin of the phylogenetic line [38,39] are other phenomena that are not supported in gradualistic evolutionary theories but are explicable within class V punctuational theories. Webster [40] reported that the frequency and extent of morphological variations in 982 trilobite species are greatest early in the evolution of the group. He has shown that “the proportion of species with at least one polymorphism drops sharply between the Middle Cambrian (75%) and Late Cambrian (8%), then rises to 40% in the Early Ordovician (coincident with the first sampling of the diverse phacopid and proetid orders), after which there is a progressive decline through the Middle Devonian (1%), interrupted only by a particularly low value (0%) in the Late Silurian. No polymorphism was recorded in character-state coding among the 23 post-Devonian species [41]”.

Change in the diversity of a clade (but not necessarily the abundance of a species) is usually asymmetrical in time; a clade quickly achieves maximum diversity and slowly goes extinct [39,42]. In addition, the speciation rate usually declines with increasing age of a clade [43,44]. Both phenomena could have a common cause, continuous irreversible freezing of more and more traits during the evolution of a clade [3]. Traits differ in resistance to transition from frozen to plastic in response to reduction of genetic polymorphism. For some traits, this is likely to happen readily, coupled with a relatively small reduction in genetic polymorphism. For others, transition from frozen to plastic is difficult or even impossible, as it requires an unrealistically long period of persistence of an unrealistically small population. On a macroevolutionary time-scale, more and more traits that are characteristic for the clade (or rather the corresponding taxon) pass into the permanently frozen state due to a universal process of sorting for stability. Stable traits (systems etc.) persist while unstable traits (systems, etc.) pass away. A stable trait is a trait coded by many genes that are interchangeable in their effect. The mutation of an allele in one locus does not result in a change in such a trait, while mutation in all the loci is highly improbable especially if, due to pleiotropy, the genes in particular loci also influence other traits. Another source of the evolutionary stability of a trait is frequency-dependent selection, particularly the steep dependence of fitness on the frequency of an allele. When the fitness of an individual decreases sharply with the increased frequency of an allele (of a particular trait), even a drastic reduction in population size cannot lead to total loss of the polymorphism in a particular locus. Due to dominance, and especially to epistatic interactions of more than two genes, the slope of fitness can be very steep. In the dominance case, the fitness of homozygotes with genotype aa could decrease at a rate proportional to the second power of the trait frequency. In the case of epistatic interactions between more than two genes, the rate could be proportionally higher. This kind of trait probably survives peripatric speciation in a polymorphic state, or polymorphism in such a trait is restored very quickly in the newly emerging species due to mutations.

In a new taxon, i.e., a clade that was named by taxonomists because of the presence of certain combination of (‘important’) traits, a relatively high proportion of species contain many apomorphic traits that could become unfrozen during standard peripatric speciation or that are relatively plastic even at the level of a species (or even of a local population). In time, more and more traits in more and more species turn to a semi-permanently or even permanently frozen state. The representatives of a particular taxon are not only less and less variable (more and more elastic – resistant to selection pressure) but also exhibit elasticity that is less and less affected by future peripatric speciations. Originally, many representatives of a taxon had the capacity to evolve new body plans after peripatric speciation. In the end, only some species retained this capacity and, even in these species, some traits had a highly limited capacity to respond to selection after peripatric speciation.

### Dead Clade Walking

This last mechanism can explain another well-known phenomenon, namely: dead clade walking. It is widely known that unexpectedly many diversified and diversifying clades that survive a period of mass extinction turn marginal or decline in the aftermath stage. Jablonski [45] wrote that “For four of the Big Five mass extinctions of the Phanerozoic, the marine genera that survived the extinction suffered about 10–20% attrition in the immediately following geologic stage, significantly greater than the losses sustained in pre-extinction stages. The stages immediately following the three Palaeozoic mass extinctions also account for 17% of all order-level losses in marine invertebrates over that interval, which is, again, significantly greater than for other stratigraphic stages (no orders are lost immediately after the end-Triassic or end-Cretaceous mass extinctions).” Such a pattern could be expected when all the representatives of a clade that survived the mass extinction were irreversibly frozen [3]. A clade depleted of all the species that can be turned plastic by peripatric speciation cannot adapt to the changing environment and would probably become extinct in the next chronostratigraphic stage.

### Cambrian Explosion

Another phenomenon that cannot be explained within the traditional gradualistic evolutionary theories is the Cambrian explosion [46,47]. All the basic animal architectures were apparently established by the close of the Cambrian explosion; subsequent evolutionary changes, even those that allowed animals to move out of the sea onto the land, involved only modifications of those basic body plans. Most probably, not only the general diversity of metazoan body plans, but also the diversity within particular phyla reached its maximum within 10–20 million years during the Cambrian , and remained stable or even decreased throughout the following 500 million years [46,48]. The number of species increased irregularly and discontinuously during the Phanerozoic; however, the number of body plans, i.e. disparity, probably decreased.

Considerable efforts have been exerted to suggest that the Cambrian explosion, a phenomenon that had no support in contemporaneous gradualistic evolutionary theories [49,50], is not in any way mysterious or that it never even occurred [51-54]. Molecular clock data based on concatenated amino acid sequences of 129 proteins from 36 eukaryotes suggest that representatives of metazoan phyla probably diverged 100–210 million years before the Cambrium [55]. (Previous molecular studies suggested an even earlier divergence time; however, the results of current multigene studies are more reliable.) Nevertheless, this molecular data is useful for tracking events of cladogenesis, but not events of anagenesis [56]. The metazoan phyla could diverge long before the Cambrian; most probably, however, their representatives had very uniform body plans until the beginning of the Cambrian when some extrinsic (ecological) or intrinsic (genetic) event probably triggered the morphological diversification of the Metazoa.

The Cambrian explosion is in accordance with predictions of class V punctuational theories. At the beginning of the evolution of the metazoan clade, many traits, even those that determine body architecture, had the capacity to turn plastic during peripatric speciations in many metazoan lineages. Therefore, both radical remodeling of body architecture as well as novel origination therein were possible in the early stages of metazoan evolution. Through time, more and more traits became permanently frozen. Most probably, different traits would lose the capacity to turn plastic in differing successions in particular phyla. Therefore, anagenetic potential faded and adaptation came to be based on modification of existent plans rather than creation of new ones. Were something, e.g. a virus or humankind, to kill all the metazoan species on Earth with the exception of a single cockroach species, classical evolutionary theories argue this species would differentiate into many new phyla with radically different body plans to exploit all the available niches. The frozen plasticity theory explicitly argues [3] that it would differentiate into many new species of cockroaches, leaving most niches empty.

### Objective Existence of Species and Genus Taxonomic Categories

The punctuational theories suggest that the taxonomic category of species, and sometimes even that of genera and higher taxa, could objectively denote the existing entity, rather than merely being a useful epistemological construct of biologists. Within any punctuational theory, a biological species can be defined as a set of individuals sharing an identical gene pool throughout the period between two speciation events. Similarly, within class III-V punctuational theories, a genus can be defined as a set of individuals sharing a common exclusive ancestor in the period between two periods of evolutionary plasticity.

## Conclusions

The picture of macroevolution postulated by most of present punctuational theories of evolution corresponds well with palaentological data and the punctuated equilibrium model of evolution [57,58] is now a generally accepted model of the evolution of multicellular life on Earth [59].

However, the punctuational models could have a very important impact on understanding, not only macroevolutionary processes, but also microevolutionary and ecological processes (see Table 2). The picture of ecological processes presented by punctuational models differs in many respects from that provided by the current textbook theory of evolution and ecology. All of these predictions of punctuational models could be tested empirically and/or analyzed in greater depth theoretically. Although the previous section mentioned a number of facts demonstrating the correctness of the punctuational models of evolution, it must be emphasized that none of them can be considered to prove it when taken alone. Alternative explanations for any of the above-mentioned facts that do not encompass the concept of punctuated evolution already exist. However, these alternative explanations are *post hoc* explanations, sometimes not very probable and sometimes rather awkward and, in addition, frequently mutually incompatible or incompatible with the currently accepted explanations of other phenomena. In contrast, the punctuational models of biological evolution were established prior to accumulation of most of the data that now confirm their validity. In my opinion the punctuational theories of evolution offer new predictions that should be tested and provides explanations for a much broader spectrum of known biological phenomena than classic gradualistic evolutionary theories.

## Reviewers' comments

### Report 1

#### Claus Wilke, The University of Texas at Austin, United States of America

Overall, this is an interesting and thought-provoking article, and I'll be happy to see it published. I have one major request, though: The author states repeatedly that it would be possible to test the various theories against each other. I think the paper would improve greatly from a section that explicitly suggests concrete tests to distinguish theories. As I was reading the paper, I was waiting for such a section, but it never came.

Author’s response: *I would like to thank the referee for encouraging comments. I included the hypotheses to be tested in particular subchapters and also summarized the predictions that may distinguish between the discussed models of evolution in Table*2*. I believe that corresponding specialists (paleontologists, ecologists, molecular biologists etc.) are more competent to suggest proper experimental designs for concrete tests of particular hypotheses. Moreover, many of these tests have already been performed; see Table*2*and the listed references.*

### Report 2

#### Pierre Pontarotti, Universite d'Aix Marseille, France

The author presents quickly the gradualist versus the punctualist theories of evolution, including his own theory (published in 1998) . The readers of this article would really need to use the two tables (included in this article) and to read the articles noted in references to really understand the differences between the different theories, which is a lot of work. Beside the field specialists I am afraid that very few people will read the article. I would like to add that the original article describing the author theory got very few citations, showing that few scientists are aware of the author theory. My advice is that the author should rewrite his article and specially the introduction on a more synthetic way.

Author’s response: *I agree with the referee that it is difficult to understand the differences between the discussed theories without careful study of the Table*1*. According to my opinion, a similar study of Table*2*(which summarizes differences in the implications of particular theories) is not necessary. Actually, the present paper was originally part of the paper “Elastic, not plastic species: Frozen plasticity theory and the origin of adaptive evolution in sexually reproducing organisms” Biol. Direct 2010, 5:2. It is therefore useful (but not necessary) for readers of the present article to also read the previous one. For the convenience of readers, I decided to include Figure*1*, summarizing the mechanism of the frozen plasticity model (the only representative of Group V punctuational theories of evolution) into the corrected version of the present paper. For a detailed understanding other punctuational theories of evolution, I recommend the excellent review by A. R. Templeton*[1]*.*

The author then go through, different evolutionary biological observations and analyses, and advocates that these observations and analyses support his evolutionary theory. This kind of approach can be criticize as the readers can argue that the author use a correlative approach to support his hypothesis. But this is the way the science goes, the readers could then found evidences that can go against the author theory and this could start a fruitful debate. I really like the idea include in the short paragraph: “objective evidence of species and genus taxonomic categories” . So maybe Genus is a real concept and not only a classification label. I encourage the author to develop this idea and to include it in the title. This idea should interest all the scientist interested in the taxonomy and evolutionary biology in general.

Author’s response: *The possibility that the Genus is a real concept and not only a classification label, is just one of 19 implications of punctuational theories of evolution discussed in the present article. It would be rather strange to include it into the title of the article. However, I changed the title of the article from, “Microevolutionary and macroevolutionary implications of punctuational theories of adaptive evolution” to “Microevolutionary, macroevolutionary, ecological and taxonomical implications of punctuational theories of adaptive evolution” and modified the list of keywords.*

### Report 3

#### David Penny (nominated by Anthony Poole), Institute of Molecular BioSciences, Massey University, North New Zealand

It is important, as the author point out, to split hypotheses up into subcomponents. This is an important aspect that is often overlooked, but in this case there is still to be a significant amount of confusion. I have recently [60] divided evolution into 20 components that can be tested individually, and so like the approach of testing components. Perhaps the first one to get in the way is the one discussed here – the so-called gradualism-punctuational debate that was introduced by William Whewell in his 1832 review of Lyell’s Principles of Geology [61] Rhodes [62] and Penny [63] have tried to set the record straight, that Darwin (and before him, Charles Lyell and James Hutton) clearly distinguished ecological and geological time scales. For example, Darwin stated that he thought “… the periods during which species have been undergoing modification, though long as measured by years, have probably been short in comparison with periods … without undergoing any change”. Thus the physical laws were the same, but the biological consequences could be variable. So if we could possibly get rid of Whewell’s confusion, we can face the gradualism/punctuated issue positively (although, we prefer to emphasize the continuity aspect that is common to both).

However, I still think that we biologists are not really asking the fundamental question; the question that Charles Lyell (Darwin’s mentor) asked about 180 years ago (although at that stage it was more about geology). The subtitle of his Principles of Geology asked simply the extent that ‘former changes to the Earth’s surface’ were referable to ‘causes now in operation’ [60]. So the question was very much about mechanisms, not about description by itself. Charles Darwin took this question, and during the voyage of the Beagle, became convinced that that Charles Lyell’s approach/question was fundamental. On his return to London, Charles Darwin joined the Geological Society (and not the Linnean nor the Zoological Societies). Similarly, his early papers were on geological topics, and he wrote three books on geology from the voyage. Darwin edited the zoological works from the voyage, but is botanical specimens were effectively lost for over a century [64]. But certainly, Darwin’s attention quickly changed from geology to more biological topics, but his interest in ‘causes now in operation’ (fortunately) did not change. It was a very mechanistic approach to evolution (though obviously limited by what was known at the time).

Author’s response: *In the present paper, I divided punctuational theories of evolution into five classes on the very basis of mechanisms responsible for the evolutionary stasis – see*[65-67]*and the Table*1*for references describing particular mechanisms in detail. A short description of the class V theory, the frozen plasticity theory, i.e. the genetic mechanisms responsible for the evolutionary stasis and the mechanism responsible for the transition from frozen to plastic state, is shown in the chapter Background. In the corrected version of the paper, these mechanisms are illustrated by the Figure*1*. Old genetic mechanisms of stasis and transition into plastic state are discussed here*[1]*and the new genetic mechanism of stasis (based on pleiotropy and frequency dependent selection) and transition into evolutionary plastic stage (based on elimination of genetic polymorphism by founder effect, bottleneck effect and genetic drift) is discussed in details in the paper*[3]*and in the book Frozen Evolution*[68]*.*

So we will now see why I am not sure if Jaroslav Flegr is asking quite the right question. If we concentrate on mechanisms we might well ask how can genes and/or proteins possibly ‘know’ that they are in a speciation phase? How do the DNA polymerases, and the DNA error-correction proteins ‘know’ that it is time to relax their abilities, and not make so many corrections because the host is undergoing speciation. How does a gene pool ‘resist’ selection? Pass, I have no idea how a ‘species’ could be so intelligent as to know it is speciating. It appears simpler at present to assume the simplest model, namely that the level of mutation is approximately (statistically) constant. Okay, there is likely to be variation with some life history parameters - we probably can’t have the combination of very long lived species combined with very high mutation rates – there might be too many mutations between parents and their offspring? But that is a mechanistic question that can be tested empirically – we don’t have to ‘believe’ anything.

Author’s response: *Probably, there are some theories of punctuational evolution based on different rate of mutations or different stringency of reparation processes during evolutionary stasis and during phase of accelerated evolution. For example, the molecular apparatus of germinal cells could recognize a phase immediately after a colonization speciation on the basis of decreased heterozygosity, and could generate new genetic polymorphism by suppressing some reparation processes. However, the genetic theories of punctuational evolution discussed in the present paper expect the same rate of mutation and reparation processes during frozen and plastic phases of existence of a species. The useful mutations are generated in the same rate, however, during the frozen state of a species, these mutations cannot spread by selection because of reasons discussed for example here*[1,3]*.*

Again, perhaps I have another concern - why focus on ‘speciation’? If there is a continuum [69] from individuals, populations, varieties, sibling species, species, species complexes subgenera, genera, etc. then is there anything really special about the species level? From an evolutionary point of view, there certainly does not appear to be anything special about the level of species – it is probably an older concept probably dating to the late 17^th^ century.

Author’s response: *I agree, there is nothing special about the level of species and a continuum from populations to species most probably exists. However, there is something very special about the peripatric speciation (and possibly also about other types of speciation such as a certain category of polyploid speciation). According to class III-V theories of punctuational evolution, these speciation events can result in the transition of a population from its frozen to plastic state and therefore can result into the origin of radically different phenotype of the new species. Of course, by other types of splitting of species (populations), the new species can also arise. However, without the transition from the frozen to the plastic state, the phenotype of representatives of new and old species are usually very similar and usually differ only by selectively neutral characters fixed by drift or by founder effect. To make my view absolutely clear: according to class III-V theories, Charles Darwin was probably right – there’s nothing special about species, the sets of individuals sharing an identical gene pool throughout the period between two speciation events – species are from men (namely from taxonomists). Conversely, there’s something very special about genera, the sets of individuals sharing a common exclusive ancestor in the period between two periods of evolutionary plasticity – genera are from God (namely from the transition of a species from the frozen to plastic state).*

So basically, I like the idea of rates of adaptation being variable, but I still think that we should focus on the mechanisms available for evolutionary change, and not just to consider the description. We assume that species are not optimally adapted to their environment. For example, for most of life we did not have flowering plants; we did not have mammals; and we did not have humans. As far as we know, there was nothing in the physical environment that precluded these groups during the last three billion years or so. Thus I appreciate Flegr’s questioning of some old dogmas, but perhaps I still want to see a more mechanistic approach to evolution.

I have given a very general report - that I hope is self-explanatory. I have tried hard to like the manuscript, but I guess I am rather too committed to the mechanisms that lead inevitably to evolution to appreciate the more descriptive approach of the author.

Author’s response: *The present paper is about implications of various models of punctuational evolution. For study of genetic mechanisms of punctuational evolution, I have to recommend the readers an excellent review by Templeton*[1]*and my previous paper in Biology Direct*[3]*and for empirical evidence see*[70-73]*and other references listed in the Table 2*.

## Competing interests

The author declares that he has no competing interests.

## Authors’ information

JF is a professor of ecology at the Department of Philosophy and History of Science, Faculty of Science, Charles University of Prague. He is an author of four books on evolutionary biology and evolutionary parasitology.
